# Identification of Mechanosensitive Genes during Embryonic Bone Formation

**DOI:** 10.1371/journal.pcbi.1000250

**Published:** 2008-12-26

**Authors:** Niamh C. Nowlan, Patrick J. Prendergast, Paula Murphy

**Affiliations:** 1 Department of Zoology, School of Natural Sciences, Trinity College Dublin, Dublin, Ireland; 2 Trinity Centre for Bioengineering, School of Engineering, Trinity College Dublin, Dublin, Ireland; Eindhoven University of Technology, The Netherlands

## Abstract

Although it is known that mechanical forces are needed for normal bone development, the current understanding of how biophysical stimuli are interpreted by and integrated with genetic regulatory mechanisms is limited. Mechanical forces are thought to be mediated in cells by “mechanosensitive” genes, but it is a challenge to demonstrate that the genetic regulation of the biological system is dependant on particular mechanical forces in vivo. We propose a new means of selecting candidate mechanosensitive genes by comparing in vivo gene expression patterns with patterns of biophysical stimuli, computed using finite element analysis. In this study, finite element analyses of the avian embryonic limb were performed using anatomically realistic rudiment and muscle morphologies, and patterns of biophysical stimuli were compared with the expression patterns of four candidate mechanosensitive genes integral to bone development. The expression patterns of two genes, Collagen X (ColX) and Indian hedgehog (Ihh), were shown to colocalise with biophysical stimuli induced by embryonic muscle contractions, identifying them as potentially being involved in the mechanoregulation of bone formation. An altered mechanical environment was induced in the embryonic chick, where a neuromuscular blocking agent was administered in ovo to modify skeletal muscle contractions. Finite element analyses predicted dramatic changes in levels and patterns of biophysical stimuli, and a number of immobilised specimens exhibited differences in ColX and Ihh expression. The results obtained indicate that computationally derived patterns of biophysical stimuli can be used to inform a directed search for genes that may play a mechanoregulatory role in particular in vivo events or processes. Furthermore, the experimental data demonstrate that ColX and Ihh are involved in mechanoregulatory pathways and may be key mediators in translating information from the mechanical environment to the molecular regulation of bone formation in the embryo.

## Author Summary

While mechanical forces are known to be critical to adult bone maintenance and repair, the importance of mechanobiology in embryonic bone formation is less widely accepted. The influence of mechanical forces on cells is thought to be mediated by “mechanosensitive genes,” genes which respond to mechanical stimulation. In this research, we examined the situation in the developing embryo. Using finite element analysis, we simulated the biophysical stimuli in the developing bone resulting from spontaneous muscle contractions, incorporating detailed morphology of the developing chick limb. We compared patterns of stimuli with expression patterns of a number of genes involved in bone formation and demonstrated a clear colocalisation in the case of two genes (Ihh and ColX). We then altered the mechanical environment of the growing chick embryo by blocking muscle contractions and demonstrated changes in the magnitudes and patterns of biophysical stimuli and in the expression patterns of both Ihh and ColX. We have demonstrated the value of combining computational techniques with in vivo gene expression analysis to identify genes that may play a mechanoregulatory role and have identified genes that respond to mechanical stimulation during bone formation in vivo.

## Introduction

It is widely accepted that there is a relationship between the morphology of skeletal structures and the mechanical forces acting upon them. Such a relationship begins in the embryo where the importance of muscle for normal bone formation has been clearly demonstrated [1],[2]; however, it is still not understood how biophysical stimuli are interpreted and integrated with the genetic regulatory mechanisms guiding bone development. Presumably gene activity within the skeletal tissues is influenced by mechanical stimulation but there is very limited information on how this might occur in the embryo. Up- and down-regulation of gene expression due to mechanical stimulation has been demonstrated under certain cell culture conditions, and these genes have been called mechanosensitive genes [3]. Most experiments revealing mechanosensitivity have placed cells under mechanical stimulation in culture and subsequently performed analyses to quantitatively compare the expression of many genes between stimulated and control cells, for example using microarray analysis (e.g., [4],[5]). Using such an approach, hundreds of potential mechanosensitive genes can be identified simultaneously; however, these experiments do not demonstrate the mechanosensitivity of a gene in an in vivo context. To establish that a gene plays a mechanoregulatory role during a particular process it is necessary to examine the sensitivity of the gene to mechanical stimulation in vivo.

It is, however, more challenging to examine candidate genes in an in vivo context. To date, the study of Kavanagh et al. [6] is unique in demonstrating a mechanoregulatory role for a gene during embryonic development in vivo by altering the mechanical environment. These authors examined the expression patterns of three signalling molecules which are implicated in regulating joint formation; growth and differentiation factor 5 (GDF-5), fibroblast growth factor-2 (FGF-2) and FGF-4 in control and immobilised chick embryonic hindlimbs and showed that joint line FGF-2 expression was diminished in immobilised limbs, while the expression of the other two genes in the joint line was unaffected. They concluded that FGF-2 has a direct mechanoregulatory role in the cavitation process. Another approach has been to use computational modelling to identify candidate mechanosensitive genes, where regions predicted to be under high mechanical stimulation are correlated with the expression of certain genes. Henderson et al. [7] used a 2-D finite element model to predict patterns of growth-related stresses and strains generated during the growth of a skeletal condensation for comparison with in vivo expression patterns of “chondrogenic genes” and “osteogenic genes”. By comparing patterns of biophysical stimuli with gene expression data from transverse sections, they proposed that predicted patterns of pressure correspond with expression patterns of chondrogenic genes and that predicted patterns of strain correspond with patterns of osteogenic genes. Their model focussed exclusively on growth related biophysical stimuli and did not, therefore, examine the effect of embryonic muscle contractions.

Considering embryonic bone formation specifically, a number of genes involved in key steps have been identified as mechanosensitive in in vitro cell culture assays [3],[8]. These include genes encoding Collagen X (ColX), Fibroblast Growth Factor receptor2 (FGFr2), Indian hedgehog (Ihh) and Parathyroid hormone-related protein (PTHrP). ColX encodes a structural protein synthesised by hypertrophic chondrocytes [9] that has been identified as playing a role in matrix mineralization [10], and was shown to be upregulated in in vitro cultures of bovine chondrocytes under cyclic tension and cyclic hydrostatic pressure [11] and in ex vivo mechanical stimulation of neonatal rabbit distal femoral condyle explants [12]. FGFr2 is a positive regulator of chondrocyte proliferation [13], and has been shown to be downregulated following in vitro four point bending of MC3T3-E1 preosteoblasts [5] and upregulated in in vitro mechanical stimulation of bone marrow stromal cells [14]. Ihh is also a positive regulator of proliferation [15], and controls the onset of chondrocyte hypertrophy primarily via PTHrP [16]. Ihh signalling from the proliferative region is necessary to induce the differentiation of the perichondrium into an osteogenic tissue from which the first osteoblasts will differentiate [15]. PTHrP signalling has been shown to negatively regulate the switch from a proliferative immature chondrocyte to a post-proliferative mature hypertrophic chondrocyte [17]. Ihh and PTHrP have been shown to be upregulated by mechanical stimulation; Ihh and PTHrP in in vivo mechanical stimulation of rat mandibular condyles [18],[19], Ihh in in vitro cyclic mechanical stimulation of embryonic chick chondrocytes [20] and PTHrP in in vitro cyclic mechanical stimulation of rat growth plate chondrocytes [21].

In this paper, we hypothesise that mechanical forces influence embryonic bone formation by regulating expression of mechanosensitive genes. To test this hypothesis, the involvement of four genes in transducing mechanical information from spontaneous muscle contractions during ossification was assessed; these are ColX, FGFr2, Ihh and PTHrP. The genes were selected for this study based on their importance for bone formation and evidence of their mechanosensitivity in vitro. Using a novel approach, the potential in vivo mechanosensitivity of these genes is initially assessed using computationally derived data on the biophysical environment. The candidate genes were first examined by correlating their expression patterns with patterns of biophysical stimuli across stages of development when ossification begins. We carried out a detailed analysis of expression of the 4 candidate genes and, by using the results of finite element analyses based on 3-D rudiment morphologies and realistic muscle loading schemes described in a previous paper [22], we could compare the complex and time-dependant patterns of biophysical stimuli induced by embryonic muscle contractions with gene expression patterns at several timepoints. To corroborate the correlations found, the direct response of both the genes and the patterns of biophysical stimuli to a perturbation in the mechanical environment in vivo were examined. If genes whose expression patterns could be shown to have altered expression patterns in a perturbed mechanical environment, then this would provide strong evidence that genes mediate a genetic regulation of the response to mechanical information during embryonic bone formation.

## Materials and Methods

### Avian Model

Morphological and gene expression analyses were carried out on the tibiotarsal rudiment in the hindlimb of the embryonic chick. Dissected embryos were staged according to the Hamburger and Hamilton (HH) system [23]. Three stages were chosen for analysis; HH30, HH32 and HH34, corresponding to roughly 6, 7 and 8 days of incubation, spanning the initiation of osteogenesis in the tibiotarsus.

### Probe Synthesis

The BBSRC (Biotechnology and Biological Sciences Research Council, U.K.) ChickEST Database (http://www.chick.manchester.ac.uk/, last accessed September 2008) and bank of Expressed Sequence Tags (ESTs) from the chick genome were used as a source of cDNA clones from which to generate specific RNA expression probes for the genes of interest. The database was searched for ESTs corresponding to each gene and two ESTs were selected for each based on confirmation of perfect alignment with the gene of interest following a Basic Local Alignment Search Tool (BLAST [24]) analysis through the National Center for Biotechnology Information (NCBI, http://www.ncbi.nlm.nih.gov/BLAST/, last accessed September 2008), and on the length of the EST and its position within the cDNA of the gene of interest. ESTs of 0.5–1.0 kb were preferred. The probe generated for ColX was produced from chEST 62e2 and aligns with nucleotides 1605–2320 on Genbank sequence ref M13496.1. The probe generated for FGFr2 was produced from chEST 699l24 and aligns with nucleotides 1967–2716 on Genbank ref NM_205319. The probe generated for PTHrP was produced from chEST 533c1 and aligns with nucleotides 68–734 on Genbank ref AB175678. The Ihh cDNA clone used for probe production was a gift from C. Tickle (Dundee) and corresponds to nucleotides 2–547 on Genbank ref NM_204957. The probe generated for Scleraxis was produced from chEST 654f15 and aligns with nucleotides 416–1109 on Genbank sequence ref NM_204253.1.

Each EST clone was sequenced to verify identity. Plasmid DNA carrying the EST of interest was linearized with appropriate restriction enzymes (EcoR1 or Not1). Antisense and sense digoxigenin-labelled RNA probes were transcribed in vitro from 1 µg of linearized plasmid using T7 and T3 promoter sites (according to insert orientation) in the pBluescript II KS+ vector (all components for in vitro transcription from Roche, Germany). DNA template was degraded by incubation of probes with RNase free DNase (Roche). The probes were then purified on G25 columns (Amersham Biosciences, USA) according to the manufacturer's instructions. Probe concentrations were determined by spectophotometry and probes were stored at −20°C.

### Sectioning

After dissection, limbs selected for in situ hybridisation were fixed in 4% paraformaldehyde (PFA) in PBS over night, and dehydrated through a series of methanol/PBT (PBT = 0.1% Triton X-100 in PBS; 25, 50, 75%; 1×10 minute) washes, followed by 2×10 minutes in 100% methanol and stored at −20°C in 30 or 50 ml tubes until needed. On the morning of sectioning, limbs were re-hydrated through a series of methanol/PBT (75, 50, 25%; 1×10 minute) washes at 4°C. After 2×10 minutes washes in PBT, excess tissue surrounding the skeletal rudiments was removed in order to give optimal sectioning performance. The specimens were embedded in 4% Low Melting Agarose/PBS (Invitrogen, UK). 80 or 100 µm sections were cut in the longitudinal direction with a vibrating microtome (VT1000S, Leica) and stored in PBS in 12-well plates.

### In Situ Hybridisation

After 2×10 minute washes in PBT, free-floating sections were treated with proteinase K (20 µg/ml in PBT) for 5 minutes at room temperature. Sections were then washed twice in PBT and fixed for 20 minutes in 0.2% glutaraldehyde/4% paraformaldehyde (PFA). Fixation was followed by washes (3×5 minutes) in PBT at room temperature, and a further 30 minute PBT wash at 55°C. The sections were then prehybridised at 55°C overnight in a hybridization solution containing 2% blocking reagent (Roche), 50% formamide, 5× SSC (Saline-sodium citrate buffer), 0.5% 3-[(3-Cholamidopropyl-[(3-Cholamidopropyl) dimethylammonio]-1-propanesulfonate (CHAPS), 500 µg/ml Heparin, 1 µg/ml Yeast RNA, 0.1% Tween 20 and 5 mM EDTA (ethylenediamine tetraacetic acid) (all components from Sigma, UK, unless otherwise stated). Antisense and sense probes were denatured at 80°C for 3 minutes and sections were then incubated at 55°C over 2–3 nights in hybridization solution containing either antisense or sense probe at minimum concentrations of 2 ng/µl.

Post-hybridization washes were carried out at 60°C as follows: 2×10 minutes in 2× SSC; 3×20 minutes in 2× SSC/0.1% CHAPS; 3×20 minutes in 0.2× SSC/0.1% CHAPS. The sections were then washed for 2×10 minutes in TNT (100 mM Tris-HCl, pH 7.5, 150 mM NaCl, 1% Tween 20) at room temperature and blocked in blocking buffer (0.1 M maleic acid, 0.15 M NaCl, 3% blocking reagent (Roche)) plus 10% goat serum overnight at 4°C. Sections were incubated overnight in fresh blocking buffer (plus 10% serum) containing a 1∶1000 dilution of anti-digoxigenin Fab fragments conjugated with alkaline phosphatase (Roche) at 4°C, with rocking. The sections were then washed (5×1 hour) at room temperature in TNT and left rocking in TNT over 2 nights at 4°C. On the day the signal was developed, sections were washed in 3 changes of NMT (100 mM Tris-HCl, pH 9.5, 100 mM NaCl, 50 mM MgCl_2_) for 15 minutes each. The chromogenic reaction was carried out in NMT containing 17.5 µg/ml 4-nitro blue tetrazolium chloride (NBT; Roche) and 6.25 µg/ml 5-bromo-4-chloro-3-indolyl-phophate (BCIP; Roche). Sections were developed in the dark at room temperature with rocking for 6–8 hours and then fixed in 4% PFA/PBS for 1 hour before mounting on slides with Aquapolymount (Polysciences, Inc).

### Immobilisation

Two sets of immobilisation experiments were performed at different timepoints; named Set A and Set B. In Set A, 120 eggs were assigned as experimental embryos, and 80 as controls, while in Set B, 100 eggs were assigned as experimental embryos, and 80 as controls. The eggs were incubated for 3 days, after which 4 ml of albumen was removed with a syringe so that the embryo would sink lower in the egg and a window could be cut in the shell without rupturing the chorioallantoic membrane. Administration of the neuromuscular blocking agent Decamethonium Bromide (DMB) [25] began at either day 5 (Set A) or day 6 (Set B) of incubation. Embryos assigned to the experimental group were treated daily with 100 µl of 0.5% DMB in sterile HBSS (Hank's Buffered Saline Solution), while control embryos were treated with 100 µl of sterile HBSS. Before administration of the drug or saline solution, movement of the embryo was observed and recorded, and dead embryos were discarded. After treatment, the window was sealed using wide plastic tape and the egg returned to the incubator. The treatment was repeated daily until the embryos were harvested at days 8, 9 and 11, corresponding to stages HH30–32 at day 8, HH32–34 at day 9 and HH35–36 at day 11.

All harvested embryos were stained to reveal cartilage and bone using Alcian Blue (cartilage) and Alizarin Red (bone) using a modification of the protocol of Hogan et al. [26], with an Alcian Blue concentration of 0.l%. After staining, the embryos were photographed, and the total length of the tibiotarsus and the length of the bone collar were measured for each specimen. The numbers of control and experimental specimens at days 8, 9 and 11 are detailed in Table 1. These parameters were analysed in the statistical package R (http://www.r-project.org/, last accessed September 2008), and standard t-tests were performed in order to determine the effect of immobilisation on the morphology of the rudiments. The right limbs of embryos harvested at day 9 were immediately removed for preparation for sectioning and subsequent in situ hybridisation to analyse the expression of candidate mechanosensitive genes. Sections were compared between control and immobilised groups to determine if the altered mechanical environment had an effect on gene expression.

**Table 1:** Number of specimens analysed per day of harvesting for control and experimental groups.

	Control Embryos	Experimental Embryos	Total
Day 8	13	23	36
Day 9	5	10	15
Day 11	30	25	55

### Finite Element Analysis

**Figure 1:**
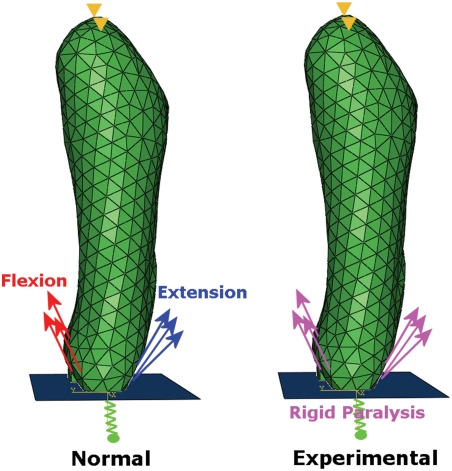
Loading schemes and boundary conditions for normal and experimental finite element analyses. In the normal analysis, muscles on the ventral aspect are active during the flexion contraction and muscles on the dorsal are active in the extension contraction. In the experimental situation, both sets of muscles are activated at the same time, at 75% of the load magnitudes of the normal situation.

As described in detail in Nowlan et al. [22] a set of finite element analyses of embryonic chick hindlimb skeletal rudiments were created for stages HH30, HH32 and HH34. At HH30 and HH32, the rudiments contain cartilage only, while the periosteal bone collar is present at the mid-diaphysis at HH34. Anatomically accurate rudiment and muscle morphologies were obtained for each stage using Optical Projection Tomography (OPT) [27], and two animals at each stage were analysed to ensure results were stage dependant rather than animal-specific. In order to characterise the biophysical environment in the absence of skeletal muscle contractions, simulations of the immobilised state were carried out and compared with the previously published patterns. Immobilisation using DMB induces rigid paralysis, where muscles are in continuous tetanus [28]. To model this situation, both ventral and dorsal muscle forces were applied simultaneously, as opposed to the situation in a normal embryo, where ventral muscles are active in flexion and the dorsal muscles in extension, as shown in Figure 1. The magnitude of the force per unit area value was also adjusted in the paralysis simulations. From the study of Reiser et al. [29], who reported the tension development in twitch and tetanic responses in normal and immobilised chick embryos, we deduced that the tetanic force response from the muscles in the immobilised chicks would be 75% of the twitch response in normal embryos. We therefore adjusted the magnitude of each of the muscle loads to 75% of the previously applied value.

## Results

### Expression Patterns of Candidate Mechanosensitive Genes

#### Collagen X

**Figure 2:**
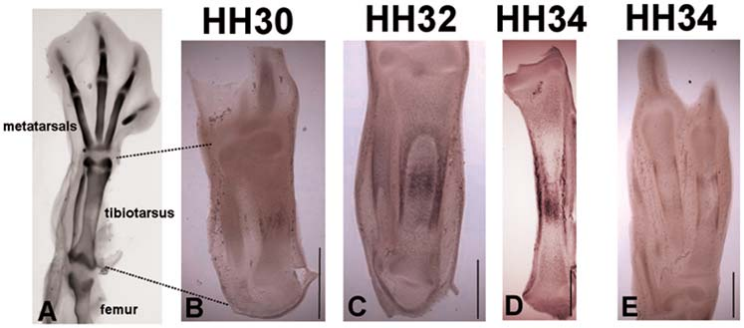
Tibiotarsal morphology and ColX expression patterns. (A) Avian hindlimb at HH34 stained with Alcian Blue to highlight cartilage, (B–D) ColX expression patterns in sections of the avian tibiotarsus at stages HH30, HH32 and HH34. ColX is expressed in the hypertrophic chondrocytes (white arrowheads) and in the perichondrium/periosteum (black arrowheads). (D) The approximate location of the bone collar is indicated with a green line. (E) ColX expression in HH34 metatarsal rudiments showing bands of expression in the perichondrium (arrows). Up is distal, down is proximal. Scale bars 1 mm.

Collagen X (ColX) expression was found in the region of hypertrophic chondrocytes in the internal cartilage of the tibiotarsal rudiments, and also in the perichondrium and periosteum (where present) at all three stages examined (Figure 2B–D). Expression in the hypertrophic region at stages HH30 and HH32 appeared as a band of increasing length at the mid-diaphysis. At stage HH34, expression in the hypertrophic zone extended further proximal and distal to the mid-diaphysis. On close examination in the hypertrophic zones at HH32 and HH34 the staining for ColX appears not to be uniform with more intense staining close to the perichondrium. While at HH30 the expression in the hypertrophic zone and in the perichondrium are localised at the mid-diaphysis, from HH32 onwards the proximo-distal extent of the perichondrial expression of ColX extends significantly beyond the expression in the hypertrophic zone. A similar expression pattern was also seen in the metatarsals of the hindlimb at stage HH34, where two distinct bands of perichondrial expression were apparent proximal and distal to the bone collar, as shown in Figure 1E, indicating that the dynamic pattern of ColX expression in the perichondrium proximal and distal to the bone collar also occurs in other limb long bones, and is not unique to the tibiotarsus.

#### FGFr2

**Figure 3 pcbi-1000250-g003:**
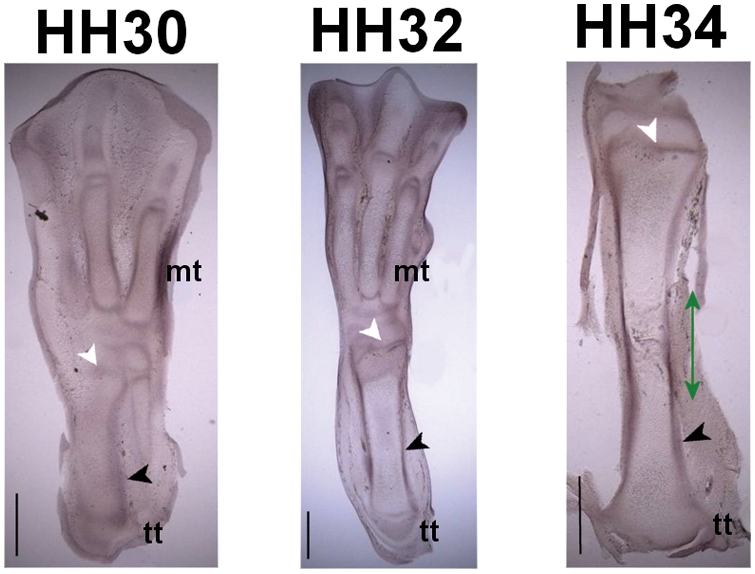
FGFr2 expression patterns in sections of the avian hindlimb at stages HH30, HH32 and HH34. Up is distal, down is proximal. tt: tibiotarsus, mt: metatarsals. The approximate location of the bone collar is indicated with a green line. FGFr2 is expressed in the perichondrium and periosteum (black arrowheads), and in the periarticular cartilage (white arrowheads). Scale bars 1 mm.

FGFr2 was found to be expressed in the perichondrium and in the periarticular cartilage of the tibiotarsus at all stages, and also in the periosteum at HH34, as shown in Figure 3.

#### Indian hedgehog

**Figure 4 pcbi-1000250-g004:**
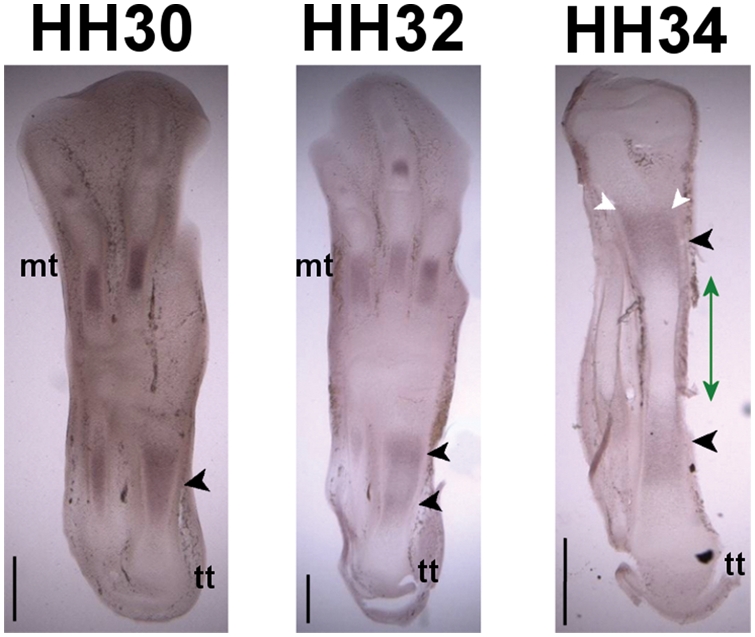
Ihh expression patterns in sections of the avian hindlimb at stages HH30, HH32 and HH34. Up is distal, down is proximal. tt: tibiotarsus, mt: metatarsals. The approximate location of the bone collar is indicated with a green line. Ihh is expressed in the hypertrophic (HH30) and pre-hypertrophic (HH30–32) zones (black arrowheads), elevated expression at the periphery highlighted with white arrowheads. Scale bars 1 mm.

Ihh was found to be expressed in bands of pre-hypertrophic chondrocytes within the cartilage core of the tibiotarsus for the three stages examined (Figure 4). At HH30, Ihh expression was found in a diffuse band along much of the length of the diaphysis. At HH32, two bands of expression were apparent proximal and distal to the diaphysis (highlighted with arrows), while at HH34, two distinct and separate bands of expression (arrows) proximal and distal to the approximate location of the newly-formed bone collar were evident. Staining in the pre-hypertrophic regions was slightly more intense towards the perichondrium at later stages (highlighted in Figure 4, HH34).

#### PTHrP

**Figure 5:**
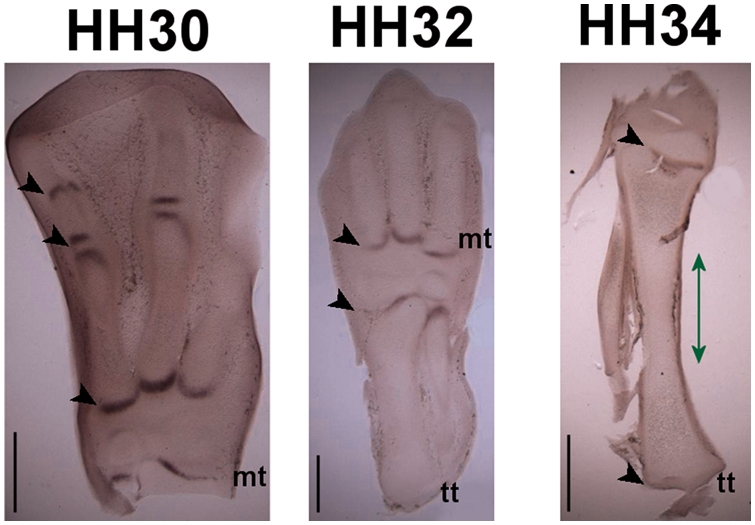
PTHrP expression patterns in sections of the avian hindlimb at stages HH30, HH32 and HH34. Up is distal, down is proximal. tt: tibiotarsus, mt: metatarsals. PTHrP is expressed in the peri-articular cartilage (arrows). Scale bars 1 mm.

PTHrP expression was evident in the periarticular regions of the rudiments of the hindlimb at stages HH30 and HH32, as highlighted with arrows in Figure 5. At stage HH34, although some PTHrP expression was present in the periarticular zone of the tibiotarsus (Figure 5), the expression appeared lower than levels present at younger stages with the staining becoming more difficult to detect.

### Correlation of Candidate Mechanosensitive Genes with Patterns of Biophysical Stimuli

**Figure 6:**
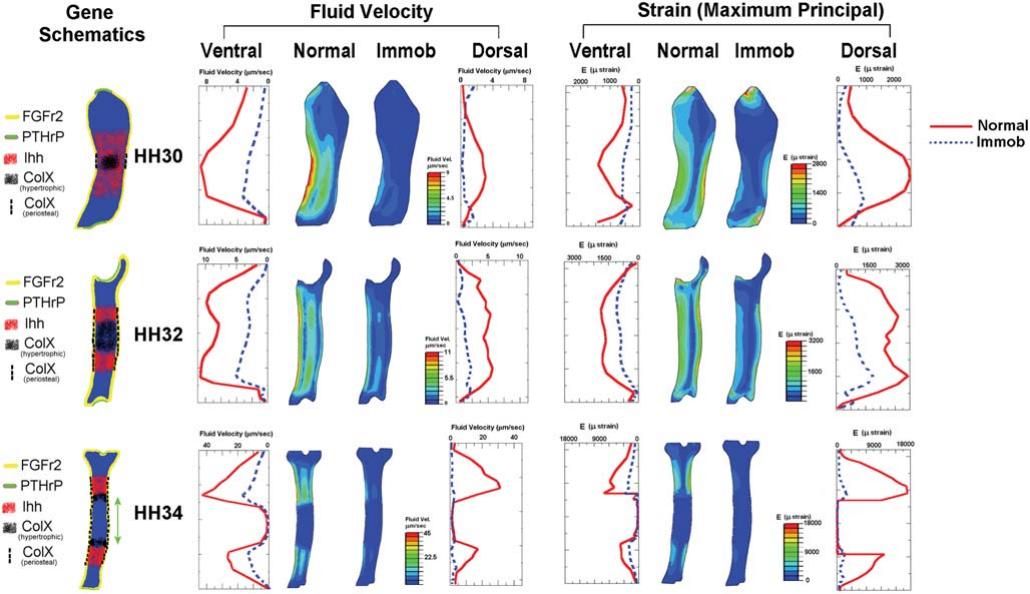
(Left) Schematic of candidate mechanosensitive gene expression patterns (Fgfr2 (yellow) expression in perichondrium and periosteum (estimated length of periosteum illustrated with green arrows). PTHrP (green) expressed in peri-articular cartilage. Ihh (red) expressed in pre-hypertrophic chondrocytes. ColX (black) expressed in hypertrophic chondrocytes (diffuse colouring), and in perichondrium/periosteum (dashed black line). (Right) Comparison of fluid velocity and maximum principal strain for normal embryos, mid-flexion (red solid line) and immobilised embryos in rigid paralysis (dashed blue line) at HH30, 32 and 34, along ventral and dorsal paths. Section shown is mid-line longitudinal section through the rudiment.

The expression patterns of ColX, FGFr2, Ihh and PTHrP illustrated in Figures 2–5 are represented schematically by stage (Figure 6) and compared with patterns of biophysical stimuli at longitudinal sections from the finite element analyses of normal (control) limbs as described in Nowlan et al. [22]. The predicted fluid velocity and maximum principal strain mid-flexion, (Figure 6) underwent distinctive changes over the three stages examined, both at the ventral and dorsal surfaces (illustrated as solid red curves), and in a longitudinal section through the middle of the rudiment (Figure 6, ‘Normal’ sections). At HH30, stimuli levels were at a high level on the perichondrium at the mid-diaphysis of the rudiment. At HH32, two concentrations of stimuli were apparent proximal and distal to the mid-diaphysis, again on the surface of the rudiment, and by HH34, these concentrations moved further apart along the length of the rudiment, proximal and distal to the newly formed bone collar [22]. Two genes showed a correlation with the patterns of biophysical stimuli; ColX and Ihh, as their expression followed patterns of events that reflect the stimuli patterns at the same stages. ColX was found to be expressed in the region of hypertrophic chondrocytes and in the region of the perichondrium where bone would soon form, spreading proximally and distally beyond the hypertrophic zone domain at the core at HH30 and HH32 and ahead of the bone collar at HH34. Therefore its surface expression demonstrated a correlation with the patterns of biophysical stimuli at each of the three stages examined (Figure 6). In the earlier stages examined, Ihh was expressed uniformly across the pre-hypertrophic zone, in one mid-diaphyseal region at HH30, and in two bands at increasing distances proximal and distal to the mid-diaphysis at later stages, the expression bands moving proximally and distally in synchrony with the biophysical stimuli at the surface. Expression of Ihh was therefore at the same longitudinal position in the rudiment as, and adjacent to, the peak levels of biophysical stimuli (Figure 6).

### Effect of Altered Mechanical Environment on Morphology, Biophyscial Environment and Mechanosensitive Gene Expression

#### Effect of immobilisation on skeletal morphology

**Figure 7:**
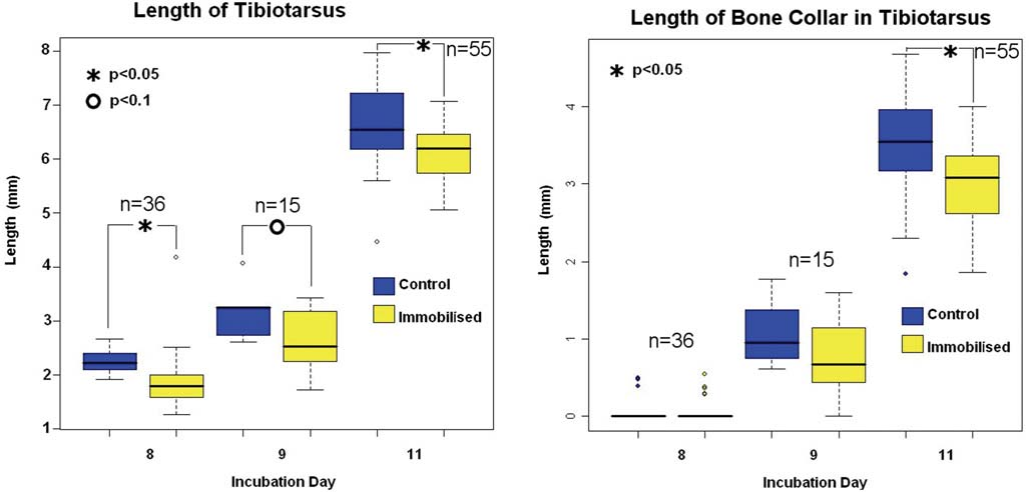
Overall length of tibiotarsus and length of bone in tibiotarsus for control (no drug treatment) and immobilised (neuromuscular blocking agent treatment) groups harvested at days 8, 9 and 11 of incubation.

In order to verify that the conditions used to alter the mechanical environment had an effect on skeletal development, the skeletons of controls and embryos treated with the neuromuscular blocking agent DMB were compared with particular focus on the tibiotarsus. The immobilisation treatment was found to have a dramatic effect on overall skeletal morphology. Treated embryos were smaller, with abnormal rib formation, joint contractures and spinal curvature (results not shown); effects which had previously been reported in other immobilisation studies [25],[30]. It was found that the tibiotarsal rudiments were significantly shorter in the immobilised group than in the control group for all days of the experiment, as shown in Figure 7a. When the length of bone (Alizarin red staining) in the tibiotarsus was measured, at day 8, most of the rudiments had not yet undergone ossification and no significant difference was found between the control and immobilised groups (Figure 7b). At day 9, there was a trend indicating less bone growth in the immobilised embryos than in controls, with no significant difference (Figure 7b). However, by day 11, a statistically significant decrease in bone length was found in the immobilised embryos (Figure 7b). No significant difference in cartilage or bone growth was found between the two sets of incubations (Set A and Set B, data not shown), demonstrating that starting the treatments on day 5 or day 6 of incubation yielded the same effect.

#### Effect of immobilisation on patterns of biophysical stimuli

When the state of rigid paralysis induced by treatment with DMB was simulated in Finite Element Analysis, dramatic differences in pattern and magnitude were observed when compared with the results from the normal skeletal rudiments, as described in Nowlan et al. [22]. Two stimuli were compared in detail, fluid velocity and maximum principal strain, as shown in Figure 6. The patterns observed previously in the normal models of one peak of stimuli levels at HH30, and two increasing proximal and distal peaks at HH32 and HH34 were not observed at all stages in the immobilised models. For example, at HH30, no peak in stimuli was observed at the mid-diaphysis, while at HH32, two very slight elevations in stimuli levels proximal and distal to the mid-diaphysis can be seen on the ventral side, while no peaks were obvious in the same regions on the dorsal side (Figure 6, HH30; HH32). A similar pattern occurred at HH34, with low peaks proximal and distal to the newly formed bone collar on the ventral aspect, with extremely low stimuli levels (in comparison to the normal situation) and no obvious peaks on the dorsal side (Figure 6, HH34). Levels of biophysical stimuli when plotted along ventral and dorsal paths are consistently lower in the immobilised rudiments (at each stage), and when we examine the levels of stimuli in a longitudinal section through the rudiment, stimuli levels are also lower throughout the breadth of the cartilage (Figure 6). As was previously reported for the normal situation [22], in the immobilised models peak stimuli levels at HH34 are significantly higher than at HH30 or HH32.

#### Effect of immobilisation on mechanosensitive gene expression

Due to the correlation with patterns of biophysical stimuli, ColX and Ihh were chosen for comparison between control and immobilized specimens at the mid timepoint of the experiment (day 9, roughly HH33). Each gene was examined in seven treated specimens and four control specimens; (of the ten experimental and five control specimens at day 9, three were damaged in the sectioning process). The analysis focussed on day 9 because, as it coincides with an early stage in the ossification process, it maximised the chances of seeing an effect on the candidate mechanosensitive genes.

**Figure 8:**
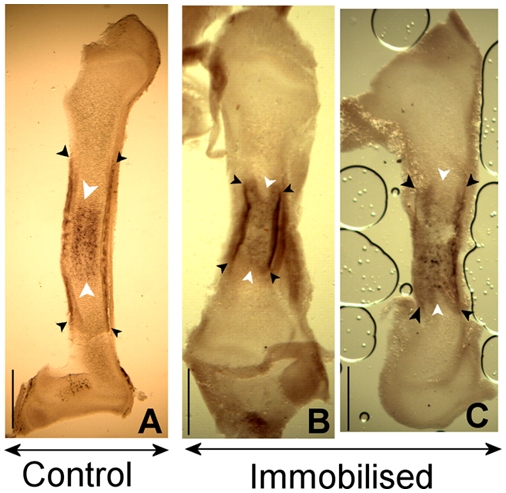
ColX expression at Day 9 in the tibiotarsus in control (A) and treated (B–C) specimens. Scale bars 1 mm. The extension of expression in the hypertrophic region is demarcated by white arrowheads; expression in the periosteum and perichondrium is highlighted by black arrow heads. B–C: ColX staining in the perichondrium is more restricted proximo-distally and does not extend beyond the hypertrophic zone.

**Collagen X.** As previously described, ColX expression is normally observed in the region of hypertrophic chondrocytes, in the periosteum (at HH34) and in the perichondrium extending proximal and distal to the hypertrophic zone. In two treated specimens, expression in the perichondrium did not extend proximally or distally beyond the hypertrophic zone as seen in untreated specimens (compare Figure 8B and 8C with 8A). Out of the seven treated specimens characterised for ColX at day 9, these differences were detectable in two specimens, while the remaining five specimens showed no differences from the expression patterns observed in controls.

**Figure 9:**
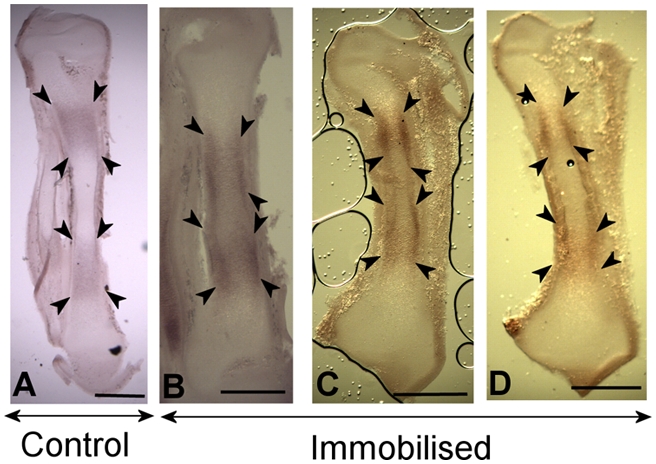
Ihh expression at Day 9 in the tibiotarsus in control (non-experimental control pictured) and immobilised specimens. Scale bar 1 mm. Expression in pre-hypertrophic chondrocytes is demarcated by arrowheads.

**Indian hedgehog.** As described above, as development progresses in the limb Ihh expression in pre-hypertrophic chondrocytes becomes slightly more intense at the peripheral edges of the domain as development progresses in the limb. This is just visible on close examination in the normal specimens at HH32 (Figure 4). Immobilisation appears to have had the effect of accelerating this localised distribution with peripheral concentrations of expression observed in three (of seven) specimens at day 9 (Figure 9), with no similar patterns observed in controls at these time points.

## Discussion

In this study, we set out to test the hypothesis that mechanical forces influence embryonic bone formation by regulating certain mechanosensitive genes. In a first analysis, the expression patterns of four genes; ColX, FGFr2, Ihh and PTHrP, were characterised and compared with patterns of biophysical stimuli. ColX and Ihh expression patterns correlated with stage-matched patterns of biophysical stimuli, whereas FGFr2 and PTHrP expression patterns did not. This identified ColX and Ihh as potential mechanosensitive genes regulating ossification in the embryo. ColX and Ihh expression patterns followed the same dynamic sequence of events as the patterns of biophysical stimuli, with one peak of expression at the mid-diaphysis at the youngest stage (HH30), and two peaks progressively more proximal and distal to the mid-diaphysis at HH32 and HH34. The ColX expression at the surface (on the perichondrium) correlates with the locations of peak biophysical stimuli also at the surface, while Ihh expression in the pre-hypertrophic cartilage is at the same longitudinal position in the rudiment as, and adjacent to, the peak levels of biophysical stimuli. In order to corroborate the hypothesis that ColX and Ihh may act as mechanosensitive genes for bone formation in the chick limb, an immobilisation assay was established, where rigid paralysis was induced with the prevention of skeletal muscle contractions. The morphological analysis of the immobilised embryos clearly demonstrated the effect of an altered mechanical environment on skeletal development, with immobilisation leading to shorter tibiotarsi and decreased bone collar formation. Finite Element Analyses of skeletal elements under rigid paralysis indicated a dramatic alteration in patterns of biophysical stimuli both in terms of stage-dependant patterns of biophysical stimuli and magnitudes of stimuli in comparison with the normal case. Aspects of the expression of ColX and Ihh indeed showed altered expression patterns following immobilisation in a proportion of specimens; (see Figures 8 and 9), corroborating their role in mechanoregulation pathways during ossification in the chick long bone.

The identification of Ihh as mechanosensitive in vivo is of particular interest since this gene has been shown to be a key regulator of bone formation in the mouse, and in particular formation of the bone collar, [15]. The elevation of expression close to the periphery of the hypertrophic zone at later stages, as described in the Results section, was precisely the aspect altered in a number of immobilised specimens with an earlier and more obvious peripheral elevation when mechanical stimulation was reduced (Figure 9) – this indicates a more complex regulation of a gene by mechanical forces than a simple up- or down-regulation on the level of expression. Alterations in Ihh expression would affect the switch from a proliferative to a pre-hypertrophic chondrocyte leading to a shorter rudiment [31], and shorter rudiments were indeed found in the treated limbs. This indicates that mechanical stimulation may play a role in regulating the position and timing of proliferation of immature chondrocytes through Ihh signalling. As the simulations of the immobilised embryos did not exhibit a specific pattern in the region of the pre-hypertrophic chondrocytes that would explain the change in the Ihh gene expression profile, these results also indicate the involvement of one or more molecules interacting with Ihh in one or more mechanoregulatory pathways. Alteration to the expression of ColX was observed in the regions predicted to have highest concentrations of biophysical stimuli (Figure 8B and 8C), where the expression in the perichondrium did not extend proximal and distal to the hypertrophic zone. The Finite Element simulations of the immobilised limbs indicated that peak stimuli levels at the perichondrium at all three stages were dramatically decreased due to rigid paralysis. It is possible that ColX may promote deposition of osteoid on the perichondrium in response to peak levels of mechanical stimulation, which would explain, at least in part, the reduced bone formation in the altered mechanical environment induced by immobilisation. Alternatively, it is possible that expression in the perichondrium does not extend beyond the hypertrophic region due to an increase in the length of the hypertrophic zone. An elevated rate of hypertrophy would lead to a shorter rudiment, as was indeed found in the immobilised specimens in this experiment. However, the altered expression profile of Ihh does not suggest an increase in the number of pre-hypertrophic chondrocytes. Therefore, it is likely that one or more other mechanoregulatory molecules are involved, and this will be a subject for future work.

In this study, there was a certain amount of variability in the effect of the neuromuscular blocking agent, and the change in expression patterns of candidate mechanosensitive genes were not seen in all immobilised (drug-treated) specimens. This variability is not unexpected since the alteration to muscle contractions is effected by exposure to a pharmaceutical agent where the response to a set dose can vary across individual specimens. A variable response was also evident when movement in the experimental embryos was quantified; while movement was clearly reduced, it was not completely removed in all specimens. However, detectable changes in gene expression were seen for two different genes in multiple specimens, showing a repeatable effect, and the statistically significant decrease in rudiment length and bone formation serves as confirmation of the immobilisation treatment as a means of altering the mechanical environment. The magnitudes of the muscle loads applied for the embryos subjected to rigid paralysis may be an overestimation, because while we have assumed the same volume of muscle in our simulations, it has been widely reported that muscle mass is reduced in immobilised embryos [32]. However, as the models are likely to overestimate the muscle forces in a completely immobilised animal, this will only strengthen our findings of the dramatic effect on the biophysical environment due to paralysis. Another limitation of this research is that late long bone ossification events are significantly different in mammals and birds [33], where the long bones of birds are formed primarily via periosteal ossification as opposed to a combination of periosteal and endochondral ossification in mammals. However, birds and mammals have the events preceding ossification in common, such as hypertrophy of the chondrocytes and formation of the periosteal bone collar, and therefore genes identified as being mechanosensitive in vivo in the chick are likely to have a similar role in the mammal.

The study presented here has revealed the alteration of gene expression as a result of mechanical stimulation. Even though we have identified the in vivo mechanosensitivity of two genes in the developing limb, we do not know what signalling cascades prompted the change in ColX and Ihh expression patterns. For example, focussing on Ihh in particular, while it has been suggested that Ihh regulates proliferation of chondrocytes through the activation of stretch activated channels by mechanical stimulation [20], it remains to be discovered what transcription factors and other intracellular molecules form the link between stretch activated channels and upregulation of the gene. As ColX and Ihh have now been demonstrated to be involved in mechanosensitive pathways in vivo at specific developmental timepoints, this opens the possibility of dissecting the upstream mechanisms involved in the response.

Many researchers have recognized the importance of the interaction between mechanical and biological factors for bone development. A range of biophysical stimuli parameters have been hypothesised to promote ossification, such as low levels of hydrostatic stress and principal strain [34], local stress and strain magnitudes [35] or low levels of octahedral shear strain and fluid velocity [36]–[39]. The results presented in this study suggest that biophysical stimuli promote ossification through the action of mechanosensitive genes, but it was not possible to determine a magnitude or level of any particular biophysical stimulus necessary for normal mechanosensitive gene expression. Although dramatic decreases in stimuli magnitudes were found between normal and immobilised simulations within stages, the immobilised stimuli magnitudes at HH34 are still higher than normal values at HH30 and HH32 (Figure 6). This may suggest that the cellular response of cells to mechanical forces in the embryo is not constant across different stages of development. It was also not possible from this study to conclude the precise nature of the mechanical stimulus, (such as strain or fluid flow), causing mechanotransduction. However, with new insight into the interactions between mechanical forces and mechanosensitive genes, computational simulations which incorporate biological and mechanobiological influences on ossification may now be further developed to include specific mechanosensitive genes. Van Donkelaar and Huiskes [40] have, in fact, already developed such a numerical model, simulating the PTHrP-Ihh control loop and its influence on growth plate development. The results of their simulation suggest that the mechanical stimulation of Ihh is likely to have a greater effect than stimulation of PTHrP, a result that was also suggested in this study, by the correlation of gene expression patterns with biophysical stimuli. Our identification of Ihh as being mechanosensitive in vivo further corroborates the findings of van Donkelaar and Huiskes [40], and demonstrates that, with the identification of other mechanosensitive genes in vivo, and the subsequent development of more complex and detailed simulations, a deeper understanding of how biophysical stimuli are interpreted and integrated with the genetic regulatory mechanisms guiding bone development can be gained.

The work presented here has provided a new insight into mechanoregulation of embryonic long bone ossification. This is the first study where finite element analyses of the embryonic limb using anatomically accurate rudiment and muscle morphologies have enabled comparison of predicted biophysical stimuli patterns with gene expression patterns, and the characterisation of the biophysical environment in the growing rudiment when skeletal muscle contractions are prevented. A means of corroborating candidate mechanosensitive genes was proposed and tested, revealing ColX and Ihh as mechanosensitive in vivo during embryonic bone formation, and also identifying them as potential key mediators in translating information from the mechanical environment to the molecular regulation of bone formation in the embryo.
